# Estimated Cardiorespiratory Fitness Is Associated With Reported Depression in College Students

**DOI:** 10.3389/fphys.2019.01191

**Published:** 2019-09-18

**Authors:** Sharon Jalene, Jennifer Pharr, Guogen Shan, Brach Poston

**Affiliations:** ^1^Department of Kinesiology and Nutrition Sciences, University of Nevada, Las Vegas, Las Vegas, NV, United States; ^2^Department of Environmental and Occupational Health, University of Nevada, Las Vegas, Las Vegas, NV, United States

**Keywords:** depression, college student, estimated cardiorespiratory fitness, diversity, university

## Abstract

Depression is a serious but treatable health issue that affects college students at an alarming rate. Improved cardiorespiratory fitness (CRF) decreases depression risk and severity but this relationship has not been fully evaluated in the college student population. Non-exercise estimated CRF (eCRF) could be used to identify students at risk for or suffering from depression. This study investigated the associations of depression and eCRF in college students. Participants (*N* = 437) completed a survey which included demographic and student-status questions, eCRF variables, and a validated depression instrument. Descriptive, chi-square, *t*-test, regression, and odds ratio analyses were employed. Depression was associated with low-fitness (*X*^2^ = 4.660, *P* = 0.031) and eCRF below age-predicted CRF (*t* = 3.28, *P* < 0.001). Predictors of increased depression included low-fitness, sexual orientation, current depression treatment, and GPA (*R*^2^ = 0.145–0.159; Adj *R*^2^ = 0.135–0.149). Odd ratio analyses determined that low-fitness increased the risk of reporting depression (β = 2.39, *P* = 0.017, 95% CI = 1.17–4.872) which remained significant when adjusted (β = 2.478, *P* = 0.017, 95% CI = 1.175–5.229). Adjusted odds ratio analyses also indicated increased risk of reporting depression for those in a sexual minority (β = 2.582, *P* = 0.001, 95% CI = 1.44,4.629) and undergoing current depression treatment (β = 2.393, *P* < 0.001, 95% CI = 2.393–13.043). High levels of fitness did not reduce the odds of reporting depression compared to age predicted CRF. A simple eCRF algorithm can be used to identify college student depression.

## Introduction

Depression is the leading cause of disability and ill-health worldwide (WHO, 2017). Symptoms of depression include persistent sadness or irritability, a lack of energy, decreased ability to concentrate, and is a predominant factor in suicide ideation (NIMH, 2018). From 2013 to 2016, depression affected 8.1% of American adults of which 80% reported a reduced capacity to perform their usual activities (Brody, 2018). Compared to the general public, college students are over three times more likely to report moderate to severe depression (8.1% vs. 30.1%, respectively) (Ibrahim et al., 2013; Brody, 2018) which was associated with poor academic performance and early withdrawal from college (Hysenbegasi et al., 2005; Thompson-Ebanks, 2016). Unfortunately, many students do not seek treatment for their symptoms (Downs and Eisenberg, 2012). For instance, the National College Health Assessment (*N* = 31,463) reported that 40.1% of respondents “felt so depressed it was difficult to function” yet only 17.9% sought professional help for their symptoms (American College Health Association, 2017). Although the National Research Council and Institute of Medicine called for prioritization of mental health interventions for at-risk individuals, effective and practical evidence-based protocols for identifying college student depression are not fully established (National Research Council Institute of Medicine, 2009; Buchanan, 2012; Ibrahim et al., 2013).

Although counseling can be effective, many university mental health centers are unable to meet the increased demand for depression treatment (Gallagher, 2012). In some cases, students report waiting several weeks to see a qualified therapist (Bruzda, 2017). Although campus clinics report being overburdened, only 25% of students with mental health disorders are willing to seek help through university programs (Downs and Eisenberg, 2012). Thus, it is reasonable to conclude that many students suffer from untreated depression and that additional antidepressant interventions must be considered. Because most campuses have gyms and other physical education programs, fitness programs aimed at improving mental health are logical options in conjunction with the promotion of school counseling.

In non-student populations, increased planned PA, commonly referred to as exercise, had large antidepressant effects (Schuch et al., 2016b) and conversely, those with low fitness levels had increased risk of developing depression (Schuch et al., 2016c). There is, however, an evidential gap regarding fitness interventions for depression in college students. This may be due in part to the well-documented challenges of assessing exercise behaviors and fitness levels (DeFina et al., 2015; Dominick et al., 2016). However, physiological responses to habitual activity-related behaviors can be reliably assessed by measuring CRF: the ability of the circulatory and respiratory systems to supply oxygen to the skeletal muscles during sustained PA (Balady et al., 2010).

The gold standard for establishing individual CRF involves measuring maximal aerobic capacity (VO_2_ max) in the laboratory by collecting and analyzing the participant’s ventilation during graded treadmill or cycling activities. Numerical results of VO_2_ max tests can be compared to age-predicted normative values or used to track changes in physical performance (Balady et al., 2010). For individuals unable to reach maximal exercise intensity during testing, VO_2_ peak is estimated using various reliable methods (Loe et al., 2016). Although valuable, these tests are inaccessible for several reasons; clinicians lack expertise, tests are time-consuming and expensive, and may pose risks in patients with musculoskeletal injuries, cardiovascular disease, or other disorders (Ross et al., 2016). Additionally, accurate results from a VO_2_ max test depend on the participant’s physical and mental ability to produce a maximal effort. Burdens of depression include poor physical functioning and a lack of interest in activity (van Milligen et al., 2011) rendering the VO_2_ max test unreliable for depressed individuals (Schuch et al., 2016c).

While not a substitute for a laboratory VO_2_ max test, fairly reliable non-exercise algorithms to estimate relative VO_2_ peak have been validated for initial assessments of CRF (Ross et al., 2016). eCRF models utilize demographic, anthropomorphic, and other variables that are commonly known by individuals and clinicians. An American Heart Association statement regarding the importance of including CRF classification in clinical practice approved non-exercise eCRF prediction equations for initial patient assessment (Ross et al., 2016). The American Heart Association statement specified an algorithm developed and validated at K. G. Jebsen Center of Exercise in Medicine (Nes et al., 2011) due to its efficacy and ease of use by clinicians and individuals (Ross et al., 2016). The Nes et al. (2014) algorithm was shown to predict cardiovascular disease related and all-cause mortality comparably with measured CRF and predict mortality independent of other known risk factors (Nauman et al., 2017a). Another longitudinal study found that persistently high eCRF and low depressive symptoms decreased mortality risk by 49% (Carlsen et al., 2018).

Although a relationship between CRF and depression has been established (Papasavvas et al., 2016; Schuch et al., 2016c), and the American Heart Association has advocated for CRF as a primary physical health indicator (Ross et al., 2016), CRF evaluations have not been suggested for the identification of those at risk for or suffering from depression. Based on the overwhelming number of college students with depression and the challenges of offering treatment, a well-validated eCRF questionnaire could be a low-cost and accessible tool to help combat this mental health crisis.

Therefore, the primary purpose of this study was to determine the associations of self-reported depression with eCRF in university students. Based on evidence regarding CRF and depression in non-student populations from original research (van Milligen et al., 2011; Aberg et al., 2012; Gubata et al., 2013; Becofsky et al., 2015), and high quality systematic reviews (Papasavvas et al., 2016; Schuch et al., 2016a, b), it was hypothesized that students with low eCRF would report increased levels of depression. Additionally, this study explored the relationships of age, sex, ethnicity, race, sexual/gender orientation, student status (class standing, student–athlete status, and student–resident status), GPA, and credit hour load with college student depression.

## Materials and Methods

### Design, Setting, and Participants

This cross-sectional study occurred during the last 3 weeks of a spring semester at a large public university in the southwestern United States. A web-based survey was promoted to students via email, classroom speaking, fliers, and at a campus-wide 4-day fitness promotion event. Participants were able to complete the anonymous survey at a time and location of their choosing. Upon completion of the survey, participants were directed to a separate electronic form to receive local fitness studios class/day passes in appreciation for their time.

### Ethics Statement

This study was carried out in accordance with the recommendations from the University of Nevada, Las Vegas (UNLV) Office of Research Integrity – Human Subjects, Biomedical Institutional Review Board (IRB). Due to the low-risk for participants, the UNLV Biomedical IRB granted exempt status for this study. By selecting “I Agree” in an online survey, all participants gave informed consent in accordance with the Declaration of Helsinki.

### Survey Instruments

#### Questionnaire Information

A self-administered questionnaire obtained information about age, height, weight, race, ethnicity, sex, gender, and sexual orientation. Student status questions included class standing, credit hours underway, GPA, and student–athlete status. From self-reported height and weight, BMI was calculated (weight (kg)/[height (m)]^2^). Questions regarding exercise habits related to frequency, intensity, and duration and were validated in a previous publication (Nes et al., 2014). Participants were also asked if they were currently taking antidepressant medication or participating in counseling/psychotherapy for the treatment of depression. These questions were followed by the PHQ-9, a validated depression survey instrument. The final questions asked for RHR and waist circumference. At the onset of the questionnaire, participants were instructed to be seated while completing the survey so they could assess their RHR after 5 min of quiet sitting.

#### Estimated Cardiorespiratory Fitness

A previously published non-exercise algorithm, derived from the cross-validation of peak oxygen uptake levels from 4,367 healthy adults, was chosen to estimate CRF (mL kg^–1^ min^–1^) (Nes et al., 2011). During the original VO_2_ max testing, 17% of the participants were unable to reach a maximal effort which resulted in the uniform classification of VO_2_ peak for reporting purposes. The constant error (*CE*) for both samples was close to zero for both sexes (Validation: Men *CE* = 0.12; Women *CE* = 0.10; Cross-validation: Men *CE* = 0.02; Women *CE* = 0.02). As well, the prediction model indicated stability between the validation (Men *R*^2^ = 0.62; Women *R*^2^ = 0.55) and the cross-validation samples (Men *R*^2^ = 0.61; Women *R*^2^ = 0.56). Although the algorithm tended to overestimate CRF for those with the lowest fitness level and underestimate those with the highest, 90.2% of women and 92.5% of men in the lowest quartiles of eCRF were correctly classified as low fitness and 91.2% of women and 93.5% of men with the highest fitness were classified as fit. Overall, these analyses indicate a valid and stable estimation of the mean VO_2_ peak. Therefore, this algorithm was found to be fairly accurate for predicting sex-specific VO_2_ peak in healthy adults in an outpatient setting (Nes et al., 2011).

Variables in the regression model include age, body composition (BMI or waist circumference), RHR, and a PA_INDEX. To obtain the PA_INDEX, questions regarding exercise frequency, duration, and intensity were scored and weighted according to a previously published index (Nes et al., 2011, 2014; Nauman et al., 2017b). The PA_INDEX correlates fairly to moderately well with measured peak oxygen uptake (*r* = 0.44 for men and *r* = 0.38 for women) (Nes et al., 2011). Although waist circumference was used to estimate body composition in the original model, an alternative algorithm using BMI was found to produce nearly equivalent results (Nes et al., 2014). Due to inconsistent self-reported waist circumference measurements, the BMI model was chosen for this study:

$$\text{Men}\left(R^{2}=0.59,\mathrm{SEE}=5.8\right):92.05-\left(0.327\mathrm{AGE}\right)-\left(0.933\,\mathrm{BMI}\right)-\left(0.167\,\mathrm{RHR}\right)+\left(0.257\,\mathrm{PA}\,\_\,\mathrm{INDEX}\right)$$

$$\text{Women}\left(R^{2}=0.57,\mathrm{SEE}=5.1\right):70.77-\left(0.244\mathrm{AGE}\right)-\left(0.749\,\mathrm{BMI}\right)-\left(0.107\,\mathrm{RHR}\right)+\left(0.213\,\mathrm{PA}\,\_\,\mathrm{INDEX}\right)$$

For each participant, the numeric result of the sex-specific eCRF algorithm was compared with the age-predicted normative CRF values for healthy adults (Nes et al., 2011). To ensure accuracy, values for individual eCRF and normative CRF were compared to results from a web-based questionnaire hosted by the authors of this eCRF model (Nes et al., 2011)^1^. The scores (eCRF) as well as the difference between the eCRF score and normative CRF values for healthy adults (DIFF) were used as variables in the analyses.

#### Depression Survey – Patient Health Questionnaire-9

The PHQ-9 is a brief, validated depression questionnaire used for screening, monitoring, and measuring the severity of symptoms in research and clinical practice (Spitzer et al., 1999). Nine questions incorporate Diagnostic and Statistical Manual of Mental Disorders, version four (DSM-IV), criteria. Participants are asked to report the frequency of problems occurring in the past 2 weeks and responses are scored from 0 to 3 (Not at All, Several Days, More than Half the Days, or Nearly Every Day, respectively). According to standard PHQ-9 evaluations, the total score is divided into the following depression severity categories: (a) scores 0–4, Minimal; (b) scores 5–9, Mild; (c) scores 10–14, Moderate; (d) scores 15–19, moderately severe; and (e) scores 20–27, Severe. Internal reliability (Cronbach’s α = 0.89) and test–retest reliability (kappa of 0.84) of the PHQ-9 were assessed as excellent (Kroenke et al., 2001). Scores ≥10 have a sensitivity of 88% for major depression and an 88% specificity for accurate diagnosis (Spitzer et al., 2014). Therefore, depression status categories were assigned as Minimal and Mild depression (MIN_DEP = PHQ-9 scores 0–9) and Moderate to Severe depression (MS_DEP = PHQ-9 scores 10–27). The PHQ-9 scores were treated as a continuous variable where appropriate. Although the data were not normally distributed, skewness equaled 1.14 and kurtosis = 0.82 falling between ±2, which allowed for a parametric approach (George and Mallery, 2010; Daniel and Cross, 2019).

### Statistical Analyses

Data were analyzed using SPSS 24 with the exception of the sensitivity analyses, which were performed using R (V 3.5.3). Descriptive characteristics of the sample were calculated. Chi square (categorical variables) and independent *t*-test (continuous variables) were used to determine significant differences between students who reported MIN_DEP and students who reported MS_DEP. Univariate regression was used to evaluate predictors of PHQ scores and included eCRF and DIFF scores as well as demographic characteristics. Demographic variables that were significant in the univariate analysis were included in the multivariate analysis of total PHQ score for eCRF and DIFF separately. Because gender, age, and BMI are components for the calculation of eCRF, they were not included in the multivariate analyses. Lastly, logistic regression was used to calculate odds ratios and adjusted odds ratios for the dichotomized depression categories as MS_DEP (yes/no). Bivariate analyses collapsed eCRF scores into Yes (Fit) and No (Fit), with those at or above their age-estimated level categorized as Yes (Fit). For odds ratio analyses, fitness was categorized as: (1) Fit (reference); (2) Low Fitness; and (3) High Fitness. Fit represented those whose eCRF was within one (±1) of their age estimated level, Low fitness was <1, and High fitness >1. Significance was set at an alpha of 0.05.

## Results

Approximately 5,000 students were informed of the opportunity to participate in the study and 520 responses were collected. Submissions from participants who did not provide answers for demographic, anthropomorphic, fitness, or PHQ-9 questions were excluded (*n* = 83).

### Description and Comparisons of the Participants

Of the 437 survey respondents, 327 were women (74.8%) and 110 were men (25.2%). The average age of the participants was 23 and ranged from 18 to 59 years (SD = 5.304). Race was reported as 34% White (*n* = 149), 22% African American/Black (*n* = 96), 22% Hispanic (*n* = 95), and 22% as multi-racial and/or other (*n* = 97). Sexual orientation data was dichotomized as straight (86%; *n* = 378) and sexual gender minority (SGM) (14%; *n* = 59). Most respondents were undergraduates (92%; *n* = 402) and were not involved in organized athletics (92%; *n* = 403). GPA ranged from 1.5 to 4.0 with a mean of 3.29 (SD = 0.49). According to the estimated fitness algorithm, 45% of respondents were at or above the age-predicted level of eCRF (*n* = 197) and were deemed as Yes (Fit). Participants reported depression severity as: (a) Minimal, 44.4% (*n* = 194); (b) Mild, 27.9% (*n* = 122); (c) Moderate, 15.3% (*n* = 67); (d) Moderately severe, 7.3% (*n* = 32); and (e) Severe, 5% (*n* = 22). Therefore, categories for analyses included MIN_DEP, 72.3% (*n* = 316) and MS_DEP, 27.7% (*n* = 121).

Chi-square analyses yielded no statistically significant differences in reported depression between males and females, class standing classifications, or student athletes vs. non-athlete status (Table 1). Participants belonging to a SGM, currently receiving clinical treatment for depression, and those were not Fit were more likely than expected to report MS_DEP.

**TABLE 1:** Results of chi-square analyses.

**Variable**	**No to minimal depression**	**Moderate to severe depression**	**Chi-square value**	***p*-value**
			
	**N (%)**	**N (%)**		
**Sex**			0.056	0.814
Male	81 (25.6)	29 (24.0)		
Female	235 (74.4)	92 (76.0)		
**Sexual *o*rientation**			12.197	<0.001
Straight	285 (90.2)	93 (76.9)		
SGM	31 (9.8)	28 (23.1)		
**Race**			8.414	0.038
White	119 (37.7)	30 (24.8)		
Black	65 (20.6)	31 (25.6)		
Hispanic	61 (19.3)	34 (28.1)		
Other	71 (22.5)	26 (21.5)		
**Class *s*tanding**			7.278	0.122
First-year	39 (12.3)	23 (19.0)		
Sophomore	69 (21.8)	26 (21.5)		
Junior	78 (24.7)	35 (28.9)		
Senior	100 (31.6)	32 (26.4)		
Graduate or other	30 (9.5)	5 (4.1)		
**Student athlete**			3.876	0.491
Yes	30 (9.5)	4 (3.3)		
No	285 (90.5)	117 (96.7)		
**In-treatment**			19.811	<0.001
Yes	9 (2.8)	18 (14.9)		
No	307 (97.2)	103 (85.1)		
**Fit**			4.660	0.031
Yes	153 (48.4)	44 (36.4)		
No	163 (51.6)	77 (63.6)		

*Bivariate analyses collapsed eCRF scores into Yes (Fit) and No (Fit), with those at or above their age-estimated level categorized as Yes (Fit). No to minimal depression = PHQ-9 score 0–9; Moderate to severe depression; PHQ-9 score 10–27. eCRF, estimated cardiorespiratory fitness; DIFF, difference between eCRF and normative age predicted CRF; GPA, grade point average; SGM, sexual gender minority.*

Table 2 presents findings from *t*-tests. Those who reported MIN_DEP were significantly more likely to have a higher eCRF score and to have a positive DIFF score than those who reported MS_DEP. Additionally, those participants who reported MIN_DEP were more likely to be older and have a higher GPA than those who reported MS_DEP.

**TABLE 2:** Results of *t*-tests.

**Variable**	**No to minimal depression**	**Moderate to severe depression**	***t*-value**	***p*-value**
				
	**Mean (SD)**	**Mean (SD)**		
eCRF	46.52 (6.9)	44.97 (7.8)	1.96	0.050
DIFF	0.5 (4.7)	−1.9(5.7)	3.28	<0.001
Age	23.1 (5.4)	22.1 (3.9)	2.04	0.042
GPA	3.3 (0.5)	3.1 (5.2)	2.99	<0.004
Credit hours	13.2 (3.4)	12.7 (3.4)	0.74	0.46

*No to minimal depression = PHQ-9 score 0–9; Moderate to severe depression; PHQ-9 score 10–27. eCRF, estimated cardiorespiratory fitness; DIFF, difference between eCRF and normative age predicted CRF; GPA, grade point average; Credit hours, number of credit hours currently underway.*

### Predictors of Reported Depression

In the univariate regression analysis, several variables were significant predictors of PHQ-9 scores (Table 3). Variables that were inversely associated with PHQ-9 scores included eCRF, DIFF, and GPA. Positive relationships with PHQ-9 scores were found for sexual orientation (SGM vs. straight) and being in-treatment for depression.

**TABLE 3:** Univariate regression to evaluate predictors of total PHQ scores.

**Variable**	**β**	**Standard error**	***t***	***p*-value**
eCRF	−0.14	0.04	−3.64	<0.001
DIFF	−0.27	0.06	−4.92	<0.001
Age	−0.10	0.05	−1.85	0.067
Sex	0.79	0.67	1.19	0.238
Sexual orientation	3.71	0.83	4.49	<0.001
GPA	−1.51	0.58	−2.60	0.009
Number of credits	−0.10	0.08	−1.23	0.219
Student athlete	0.94	0.81	1.17	0.236
In-treatment	6.77	1.16	5.85	<0.001
Resident student	−0.39	1.16	−0.34	0.737

*eCRF, estimated cardiorespiratory fitness; DIFF, difference between eCRF and normative age predicted CRF; GPA, grade point average.*

Table 4 presents the results of multivariate regression analysis examining eCRF and DIFF as predictors of the PHQ-9 scores. Both eCRF and DIFF were significant predictors of PHQ-9 scores in their respective models. Variables that were positively associated with PHQ-9 scores in both the eCRF and DIFF models were belonging to a SGM, being in-treatment for depression, and belonging to the Hispanic race. An inverse association was found for GPA in the eCRF model. Sensitivity analyses were performed to assess the robustness of the multivariate linear regression models to evaluate the relationship between the total PHQ-9 score and eCRF. Monte Carlo simulations were used to generate data from normal distributions with mean of the estimated eCRF from the BMI model and the standard deviation of SEE. Different SEE values were utilized for Men (SEE = 5.8) and Women (SEE = 5.1). The simulation captured the uncertainty of eCRF by simulating data from normal distributions. To evaluate the distribution of the *p*-value for eCRF in the multiple linear regression models, 10,000 simulations were conducted. The range of these *p*-values was: 0.000015 to 0.743, with the medium *p*-value of 0.021 as compared to the *p*-value without considering uncertainty in Table 3 (0.005). A sensitivity analysis was also performed to evaluate the relationship between the total PHQ-9 score and DIFF. The *p*-value for DIFF without considering uncertainty was 0.000088 (reported as <0.001 in Table 3). The median *p*-value from 10,000 Monte Carlo simulations was 0.0073, with a range of 0.00000009 to 0.82. After including the uncertainty of eCRF in the multiple linear regression models, the estimated *p*-values were higher, but were still significant at the 0.05 level.

**TABLE 4:** Multivariate regression to evaluate eCRF and DIFF as a predictor of total PHQ scores.

	**Variable**	**β**	**Standard error**	***t***	***p*-value**
Model 1: eCRF *R*^2^ = 0.145 Adjusted *R*^2^ = 0.135	Constant	15.026	2.405	6.248	<0.001
	eCRF	−0.105	0.037	−2.847	0.005
	Sexual orientation	3.150	0.797	3.953	<0.001
	GPA	−1.332	0.553	−2.410	0.016
	In-treatment	6.221	1.122	5.545	<0.001
	Hispanic	0.804	0.586	1.371	0.171
Model 2: DIFF *R*^2^ = 0.159 Adjusted *R*^2^ = 0.149	Constant	9.145	1.887	4.847	<0.001
	DIFF	−0.213	0.054	−3.959	<0.001
	Sexual Orientation	2.956	0.793	3.727	<0.001
	GPA	−1.036	0.557	−1.859	0.064
	In-treatment	6.306	1.112	5.672	<0.001
	Hispanic	0.692	0.582	1.189	0.235

*eCRF, estimated cardiorespiratory fitness; DIFF, difference between eCRF and normative age predicted CRF; GPA, grade point average.*

### Estimated Fitness and Risk for Depression

Odds ratios and adjusted odds ratios for fitness and MS_DEP depression are presented in Table 5. Those whose eCRF scores fell within ±1 of their age-estimated value (Fit) were used as the reference group. Individuals with Low fitness were nearly two and one-half times more likely to report MS_DEP compared to those who were Fit. These results remained relatively stable when adjusted for sexual orientation but increased substantially for participation in treatment for depression. When compared to the Fit category, High fitness individuals did not have a significant difference in likelihood of reporting MS_DEP.

**TABLE 5:** Odds ratios and adjusted odds ratios for fitness and moderate- severe depression.

**Variables**	***p*-value**	**β**	**95% CI**	
			
	**REF**	**REF**	**REF**	**REF**
**Odds ratios**				
*Fitness*				
Fit				
High fitness	0.324	1.451	0.692	3.041
Low fitness	0.017	2.387	1.170	4.872
**Adjusted odds ratios**				
*Fitness*				
Fit				
High fitness	0.304	1.501	0.692	3.257
Low fitness	0.017	2.478	1.175	5.229
*Sexual orientation*				
Straight				
SGM	0.001	2.582	1.440	4.629
*In-treatment*				
Not in treatment				
In treatment	<0.001	5.587	2.393	13.043

*Fitness was categorized as: (1) fit (reference); (2) low fitness; and (3) high fitness. Fit represented those whose eCRF was within ±1 of their age estimated level, low fitness was <1, and High fitness >1. CI, confidence interval; SGM, sexual gender minority.*

## Discussion

The primary purpose of this study was to determine the relationship of depression with eCRF in university students. We also explored the relationship between depression and student demographic variables. The main finding of this study suggests that fitness, as estimated by a non-exercise CRF algorithm from Nes et al. (2011), is associated with self-reported depression in college students. We also found significant relationships between reported depression and sexual orientation, ethnicity, depression treatment via counseling or antidepressant medication, GPA, and age.

### Depression and eCRF

College students with low eCRF were significantly more likely to report depression than students with fitness levels within ±1 of their age-predicted CRF. Additionally, fitness levels were predictive of PHQ-9 scores. This is the first cross-sectional study to evaluate the relationship between depression and a non-exercise eCRF algorithm. However, our findings are supported by evidence from prospective studies conducted in non-student populations using laboratory VO_2_ max assessments (Aberg et al., 2012; Gubata et al., 2013; Becofsky et al., 2015). For instance, healthy young adults in U.S. Army training (*N* = 11,369) with poor CRF had a 36% higher incidence of mental disorders, including moderate depression (Gubata et al., 2013). Additionally, a large population study of Swedish adults (*N* = 1,117,292), low CRF at age 18 was associated with an increased risk for serious depression later in life (HR = 1.96, 95% CI = 1.71–2.23) (Aberg et al., 2012). Finally, participants in the Aerobics Center Longitudinal Study completed a maximal exercise treadmill test at a baseline examination sometime between 1979–1998 and later evaluated for depression (Becofsky et al., 2015). Compared with women with the lowest levels of CRF, women in the two highest categories of fitness had 59 and 52% lower odds, respectively, of developing depression than their unfit counterpart (Becofsky et al., 2015). Men in the two highest CRF rankings had 40–46% lower odds of reporting depressive symptoms in the follow-up survey (Becofsky et al., 2015).

Surprisingly, our findings revealed that depression levels were not significantly different between highly fit individuals and those who met their age-predicted CRF. Although evidence on this topic varies, moderate rather than vigorous levels of PA have been recommended for antidepressant effects (Craft and Perna, 2004). Similar to our results, analyses from the Aerobics Center Longitudinal Study revealed that women with the next-to-highest level of CRF had lower odds of reporting depression than those with the highest level of fitness (Becofsky et al., 2015). Conversely, an inquiry aimed at providing a dose response relationship reported a linear, positive relationship between levels of PA and good mental health (Hamer et al., 2009). It is important to interpret these findings with caution as the eCRF algorithm chosen for this investigation tended to over and underestimate fitness for those at the lowest and highest levels, respectively. Although 90.2–93.5% of the original validation study participants’ eCRF were correctly classified at or near their measured CRF (Nes et al., 2011), it is possible the algorithm underestimated eCRF for the highest fit participants in this study which could explain this finding.

### Depression in Diverse Student Populations

Consistent with previous research on sexual minority youth (Marshal et al., 2013) and first-year college students (Riley et al., 2016), we found that students in the SGM group reported significantly higher rates of depression when compared to those who self-identified as heterosexual. We also found that Hispanic race was associated with and predictive of higher levels of depression. There are inconsistencies in the literature on the relationship between ethnicity and mental health in college students. For example, one report stated that Latino/Latina students experienced higher levels of mental distress at a predominantly Caucasian university (Arbona and Jimenez, 2014) yet another found no difference in depressive symptoms between student minority ethnic groups (Visser et al., 2013). As it is crucial for university stakeholders to identify student-groups who may have increased risk for depression, lines of inquiry regarding racial and sexual diversity should remain a priority.

### Depression and Age, Participation in Depression Treatment, and GPA

Although we found some evidence that younger age was associated with higher levels of depression, further analyses did not support this conclusion. This may be due in part to the homogeneity of age in our sample. The mean age of those who reported no depression was 23 years and those with moderate to severe depression was 22.1 years. Being in treatment via counseling or antidepressant medication was one of the strongest predictors of depression. Also, self-reported GPA was lower in students with depression and low GPA was predictive of increased depression. Previous explorations regarding the relationship between academic performance and mental health have yielded mixed results. A study conducted at one university reported that diagnosed depression resulted in a 0.49 reduction in GPA (Hysenbegasi et al., 2005). Findings from another institution revealed that 44.3% of the undergraduate students perceived a reduction in academic performance due to functional impairment from mental health distress (Eisenberg et al., 2007). However, a review of the literature on this topic concluded that depression was not significantly correlated with GPA (Richardson et al., 2012). Thus, the relationship between mental health and academic performance in college students is not clarified in the literature.

### Limitations and Suggestions for Future Research

The main limitation of this study was the reliance on self-reported data. The participants may have under or over reported information if they answered with what they perceived to be socially desirable responses (Adams et al., 1999). Although the distribution of a self-administered web-based survey is a common practice, definitive conclusions should include evidence from well-designed clinical trials. Future studies could include clinical anthropomorphic measurements, the use of university student records, and a clinical assessment of depression through face to face meetings with a qualified mental health professional. Indeed, VO_2_ max testing in the laboratory could further validate our findings, but our primary purpose was to examine the usefulness of a non-exercise eCRF model to identify students at-risk for or experiencing depression. As well, the PHQ-9 is not a substitute for a clinical determination of depression and should only be considered a self-reported estimation of a 2-week occurrence of depressive symptoms. Additionally, most respondents were female and from science related departments which may have resulted in self-selection bias. Similar projects should include recruitment efforts via university-wide email announcement systems to ensure a representative sample. Finally, data collection occurred at the end of a spring semester when students may have been experiencing higher than usual distress and reduced time to exercise. Although this allowed insight into the relationship of fitness to depression at a time of apparent higher stress, a longitudinal design or cross-sectional studies at different points during the semester could provide additional information.

## Conclusion

The problem of student depression in higher education is a major public health issue that requires immediate attention. Our study provides evidence to support the use of a non-exercise eCRF algorithm to identify students at-risk for or experiencing depression. For those with low CRF, exercise-based interventions could be implemented independently or coupled with counseling at university mental health clinics. The same eCRF assessment could be used to monitor improvements in fitness. Most universities already offer recreation centers, gyms, and fitness classes rendering this a simple. low-cost, accessible intervention to improve mental health among college students.

## Ethics Statement

This study was carried out in accordance with the recommendations from the University of Nevada, Las Vegas (UNLV) Office of Research Integrity – Human Subjects, Biomedical Institutional Review Board (IRB). Due to the low-risk for participants, the UNLV Biomedical IRB granted exempt status for this study. By selecting ‘I Agree’ in an online survey, all participants gave informed consent in accordance with the Declaration of Helsinki.

## Author Contributions

SJ designed and implemented the survey. SJ, JP, and GS performed the analyses. JP and GS prepared the tables and wrote the “Results” section. SJ wrote the manuscript under with assistance from JP, GS, and BP. BP supervised the project.

## Conflict of Interest Statement

The authors declare that the research was conducted in the absence of any commercial or financial relationships that could be construed as a potential conflict of interest.

## Footnotes

1 https://www.worldfitnesslevel.org/

## Abbreviations

BMI: body mass index
CRF: cardiorespiratory fitness
DIFF: the difference between estimated cardiorespiratory fitness and a normative value based on age
eCRF: estimated cardiorespiratory fitness
GPA: grade point average
MIN_DEP: PHQ-9 score 0–9
MS_DEP: PHQ-9 score 10–27
PA: physical activity
PA-INDEX: physical activity index
PHQ-9: Patient Health Questionnaire 9
RHR: resting heart rate.
